# Development and validation of a clinical nomogram for predicting 30-day in-hospital mortality in children with moderate-to-severe traumatic brain injury

**DOI:** 10.3389/fped.2026.1754122

**Published:** 2026-06-08

**Authors:** Yi Zhong, Yuchen Liu, Mingyang Huang, Rongting Zhang, Ruxuan Zhou, Yongjun Xiang, Yuan Bin, Tianquan Yang, Yong Han, Min Chen, Hangzhou Wang

**Affiliations:** Department of Neurosurgery, Children’s Hospital of Soochow University, Suzhou, Jiangsu, China

**Keywords:** death, nomogram, outcome, risk prediction, traumatic brain injury

## Abstract

**Background:**

Traumatic brain injury is a major cause of death and disability in children, and early identification of high-risk cases is critical for improving clinical outcomes.

**Objective:**

This study aimed to develop and validate a clinical prediction model to estimate the 30-day in-hospital mortality in pediatric patients with moderate-to-severe traumatic brain injury (msTBI).

**Methods:**

A retrospective analysis was conducted on 289 pediatric patients admitted with msTBI. Independent risk factors were identified using the least absolute shrinkage and selection operator regression and multivariable logistic regression analysis to construct a clinical nomogram. Model performance was assessed using ROC curves, bootstrap validation, and decision curve analysis.

**Results:**

The median age of the cohort was 5.17 (IQR, 2.75–9.33) years. There were 101 females and 188 males. Four independent predictors were identified: Glasgow Coma Scale score, lactic acid, albumin, and trauma-induced coagulopathy. The model showed AUC of 0.898 (95% CI: 0.896, 0.899) and good agreement between predicted and observed outcomes. Hosmer-Lemeshow test yielded a non-significant *P*-value (*P* = 0.475), supporting good model calibration. Clinical decision analysis demonstrated that the threshold probability ranged from 0 to 0.95.

**Conclusion:**

This study developed a reliable clinical tool to predict 30-day in-hospital mortality in children with msTBI. It may support early risk stratification and assist clinicians in making informed treatment decisions.

## Introduction

Traumatic brain injury (TBI) remains a critical global health challenge in pediatric populations, accounting for significant mortality and long-term functional impairment (1, 2). With over 3 million children affected annually worldwide, the clinical and socioeconomic burden of TBI demands urgent attention (3). The number of pediatric patients with TBI in China increased each year at an average annual rate of 1.84%, whereas 62.80% of them are classified as moderate-to-severe traumatic brain injury (msTBI) with substantially elevated risk of mortality (4). The high burden of severe cases underscores the critical need for tools that can reliably predict the risk of death in the early clinical stage.

Numerous studies have concentrated on the several prognostic factors for pediatric TBI patients, such as Glasgow Coma Scale (GCS) score, hyperglycaemia, leukocytosis, hypotension, midline shift, and subarachnoid hemorrhage (5–9). However, significant heterogeneity across studies has led to inconsistent findings, with limited clinical consensus regarding definitive predictors (10). Although the given prediction models have contributed substantially to clinical decision-making, their clinical utility is constrained by an inability to serve as a direct risk assessment tool.

At present, the nomogram, one of the clinical prediction tools, is investigated to predict neurological outcomes in a variety of conditions. Oearsakul et al. developed and validated a nomogram for predicting 6-month follow-up outcomes in pediatric TBI patients and reported that the tool had an area under the receiver operating characteristic curve (AUC) of 0.931 (11). Zhu et al. proposed a clinical nomogram for predicting early mortality among children with moderate or severe TBI, with concordance index values of 0.918 (12). While these nomograms demonstrate promising discriminatory performance, the limited cohort size (approximately 100 cases each) may constrain the statistical power and increase the risk of model overfitting (13). Therefore, in the present study, a nomogram was developed and validated for predicting 30-day in-hospital mortality in children with msTBI, incorporating demographic, clinical, radiographic, and laboratory parameters from an expanded cohort.

## Material and method

### Study design and population

This study retrospectively included patients diagnosed with msTBI who were admitted to the Children's Hospital of Soochow University between June 2015 and December 2024. All participant data were obtained from the institutional electronic medical record system. The study was approved by the Ethics Committee of the Children's Hospital of Soochow University (per the Declaration of Helsinki; Approval No. 2024CS102). Given the purely retrospective and observational nature of the study, written informed consent was formally waived. This study was conducted following the TRIPOD statement for reporting of prediction model development and internal validation (Type 1b study).

**Figure 1:**
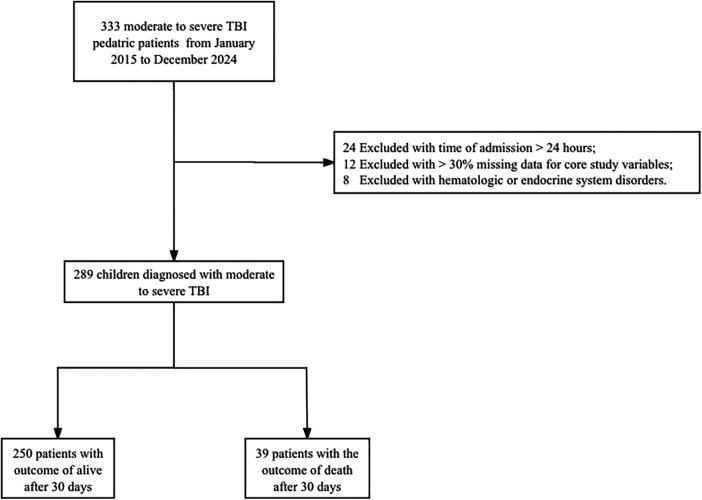
Flow chart of the study. abbreviation: TBI, traumatic brain injury.

The inclusion criteria are as follows: (1) Patients admitted within 24 h post-injury; (2) Patients presenting for initial evaluation and management at our hospital without prior intervention at other institutions; (3) Patients aged <18 years. The exclusion criteria include: (1) Patients with time of admission after injury more than 24 h; (2) Patients with a documented history of hematologic or endocrine system disorders; (3) Patients with > 30% missing data for laboratory variables. Figure 1 depicts the patient selection flowchart outlining the study design.

### Data collection and definition

In this study, all clinical variables were retrospectively extracted from the electronic records of the Children's Hospital of Soochow University. Demographic variables included age, sex, weight, and gestational age at birth. Admission trauma characteristics comprised time from injury to hospital, systolic blood pressure, and diastolic blood pressure. The Pediatric Trauma Score (PTS), a validated injury severity tool for children (14), was calculated for each patient based on patient size, airway, systolic blood pressure, central nervous system status, open wounds, and skeletal fractures. The Glasgow Coma Scale (GCS) was used to assess neurological status, with scores ranging from 3 to 15. For children under 2 years of age, the Pediatric GCS (pGCS) was employed, which adapts the verbal response component for preverbal children while preserving the same scoring range (15). Laboratory biomarkers encompassed white blood cell count, Glucose, Lactate Dehydrogenase (LDH), Lactic Acid (LAC), albumin, Blood Urea Nitrogen (BUN), Serum Creatinine (Scr), and Uric Acid (UA). Prothrombin time (PT)/international normalized ratio (INR),activated partial thromboplastin time (aPTT), platelet count, and fibrinogen were collected from the first 72 h' Routine coagulation tests. Head CT images underwent independent review by two board-certified neurosurgeons, with inter-rater disagreements resolved by consensus. To quantify the severity of radiological injury, Rotterdam CT scores were calculated based on admission head CT images (16, 17). The Rotterdam score was derived from four components: (1) basal cistern status (0 = normal, 1 = compressed, 2 = absent), (2) midline shift (0 = ≤5 mm or none, 1 = >5 mm), (3) epidural hematoma (0 = present, 1 = absent), and (4) intraventricular hemorrhage or traumatic subarachnoid hemorrhage (0 = absent, 1 = present). The total score (range 1–6) was calculated as the sum of these four components plus 1. Due to the limited number of patients with higher scores, the Rotterdam score was dichotomized as <4 vs. ≥4 for analysis (18). Extracranial injuries, including long bone fractures, pelvic fractures, thoracic trauma, and abdominal organ injury (categorized as no injury, contusion, or injury by rupture), were identified from radiology reports and medical records by the research team. Recorded interventions included neurosurgical operation and transfusion requirements.

According to the WHO guideline, anemia was diagnosed based on hemoglobin levels at admission and the patient's age (19). Trauma-induced coagulopathy (TIC) was diagnosed using age-specific laboratory thresholds based on 95% reference ranges of INR, PT, and platelet count (20). Hypotension was defined based on age-stratified normative values for hemodynamic parameters (21).

### Statistical analysis

Continuous data with a non-normal distribution are presented as median with interquartile range (IQR), while categorical variables were reported as frequency (%). Intergroup comparisons were performed using univariate analyses: Student's t-test for continuous variables and Pearson's chi-square test for categorical variables. To address collinearity and identify core predictors, variable selection was conducted through a two-stage approach: (1) Least Absolute Shrinkage and Selection Operator (LASSO) regression; (2) multivariable logistic regression incorporating variables retained by LASSO, adjusted for clinically relevant confounders.

To evaluate potential nonlinear relationships, restricted cubic splines (RCS) were fitted for continuous predictors. Significant nonlinear terms identified through RCS analysis were retained in subsequent modeling. Collinearity was assessed during model development using variance inflation factors (VIF), with VIF >5 indicating substantive collinearity. A multivariable logistic regression analysis was performed to construct the predictive model and develop a nomogram. Model discrimination was quantified by the AUC, and internal validation was conducted using the bootstrapping method (resampling = 1,000). Calibration was assessed using the Hosmer-Lemeshow goodness-of-fit test (df = 8), with *p* > 0.05 indicating adequate fit. Clinical applicability was evaluated via decision curve analysis (DCA) across probability thresholds.

To compare the baseline characteristics between isolated TBI and polytrauma patients, we performed a subgroup analysis. Additionally, to assess whether polytrauma status confounded our primary findings, we conducted a sensitivity analysis by adding polytrauma as a covariate to the multivariable logistic regression model.

Based on the method of Peduzzi et al. (22), with 39 mortality events and 4 candidate predictors (EPV = 9.75), the study had approximately 80% power to detect an odds ratio of 2.5 for a binary predictor with moderate prevalence, assuming a two-sided alpha of 0.05.

Statistical analyses were performed using SAS (version 9.4; SAS Institute Inc.) and R (version 4.2.0; R Foundation for Statistical Computing). *P* < 0.05 was considered statistically significant.

## Result

### Baseline characteristics

During the study, a total of 333 patients diagnosed with msTBI were admitted to the Children's Hospital of Soochow University. After excluding 44 patients for various reasons (Figure 1), data from 289 patients were included in the analysis. Among the remaining participants, 39 patients unfortunately lost their lives while 250 survived for at least 30 days. The median age of the cohort was 5.17 (IQR, 2.75–9.33) years. There were 101 females and 188 males. Children injured as a result of traffic accidents made up 48% of the cohort. The demographic and clinical characteristics of the study participants are summarized in Table 1.

**Table 1:** Demographic and clinical characteristics of children with msTBI.

Character	Total (*n* = 289)	Survival group (*n* = 250)	Mortality group (*n* = 39)	*P*
Gender, *n* (%)				0.963
Female	101 (35)	88 (35)	13 (33)	
Male	188 (65)	162 (65)	26 (67)	
Age (years)	5.17 (2.75, 9.33)	5.17 (2.92, 9.31)	4.83 (2.34, 9.46)	0.722
Weight (kg)	19 (14, 30)	20 (14, 31.5)	18.25 (13.12, 25)	0.489
Time from injury to hospital (hours)	3 (2, 4)	3 (2, 4)	2 (2, 3)	0.118
Mechanism of injury, *n* (%)				0.242
Traffic accident	138 (48)	124 (50)	14 (36)	
Fall off	132 (46)	109 (44)	23 (59)	
Other	19 (7)	17 (7)	2 (5)	
PTS, *n* (%)				**<0**.**001**
≤5	109 (38)	81 (32)	28 (72)	
＞5	180 (62)	169 (68)	11 (28)	
GCS score, *n* (%)				**<0.001**
<8	151 (52)	113 (45)	38 (97)	
≥8	138 (48)	137 (55)	1 (3)	
Rotterdam CT scores, *n* (%)				**0**.**332**
<4	248 (86)	217 (87)	31 (79)	
≥4	41 (14)	33 (13)	8 (21)	
Blood transfusion, *n* (%)	148 (51)	118 (47)	30 (77)	**0**.**001**
Neurosurgical operation, *n* (%)	155 (54)	132 (53)	23 (59)	0.585
Skull fractures, *n* (%)	212 (73)	186 (74)	26 (67)	0.411
Epidural hematoma, *n* (%)	110 (38)	102 (41)	8 (21)	**0**.**024**
Subdural hematoma, *n* (%)	92 (32)	76 (30)	16 (41)	0.254
Subarachnoid hemorrhage, *n* (%)	164 (57)	135 (54)	29 (74)	**0**.**027**
Intraventricular hemorrhage, *n* (%)	30 (10)	21 (8)	9 (23)	**0**.**01**
Diffuse axonal injury, *n* (%)	35 (12)	22 (9)	13 (33)	**<0.001**
Brain contusion, *n* (%)	118 (41)	99 (40)	19 (49)	0.367
Long bone fractures, *n* (%)	57 (20)	45 (18)	12 (31)	0.099
Pelvic fractures, *n* (%)	39 (13)	29 (12)	10 (26)	**0**.**033**
Thoracic trauma, *n* (%)	135 (47)	113 (45)	22 (56)	0.257
Abdominal organ injury, *n* (%)				**<0.001**
NO	224 (78)	203 (81)	21 (54)	
Contusion	51 (18)	39 (16)	12 (31)	
Injury by rupture	13 (4)	7 (3)	6 (15)	
TIC, *n* (%)	163 (56)	125 (50)	38 (97)	**<0.001**
Anemia, *n* (%)	212 (73)	178 (71)	34 (87)	0.057
Hypotension, *n* (%)	122 (48)	98 (45)	24 (67)	**0**.**024**
Glucose (mmol/L)	7.5 (6.1, 10.4)	7.3 (6, 9.6)	12.3 (7.05, 19.6)	**<0.001**
WBC ( × 10⁹/L)	17.72 (12.9, 22.77)	17.74 (12.91, 22.75)	16.46 (13, 22.86)	0.571
LDH (U/L)	573.2 (375.5, 998.2)	550.05 (366.25, 901.32)	949.5 (595.78, 1723.33)	**<0.001**
LAC (mmol/L)	2.7 (1.7, 4)	2.5 (1.6, 3.9)	4.1 (3, 6.05)	**<0.001**
Albumin (g/L)	38.4 (33.55, 42.2)	39 (34.92, 42.7)	29.55 (24.38, 38.25)	**<0.001**
BUN (mg/dL)	4.63 (3.77, 5.77)	4.64 (3.76, 5.76)	4.52 (3.8, 5.93)	0.929
Scr (*μ*mol/L)	31 (23, 41.68)	30 (23, 39.25)	41.55 (31.25, 57.5)	**<0.001**
UA (μmol/L)	284.55 (238.4, 357.65)	278.05 (237.57, 344.75)	363.25 (276.12, 433.92)	**0**.**002**

PTS, Pediatric Trauma Score; GCS, Glasgow Coma Scale; TIC, trauma-induced coagulopathy; WBC, white blood cells; LDH, lactate dehydrogenase; LAC, lactic acid; BUN, blood urea nitrogen; Scr, serum creatinine; UA, uric acid.Bold values indicate statistical significance.

Significant differences were observed between the survival and mortality groups in both the PTS and GCS score. The mortality group exhibited lower PTS and GCS score, indicating more severe trauma and neurological impairment (both *P* < 0.001). Additionally, the mortality group had significantly higher incidences of epidural hematoma, subarachnoid hemorrhage, diffuse axonal injury and pelvic fractures compared to the survival group (all *P* < 0.05). The rates of blood transfusion, abdominal organ injury, TIC, and hypotension were also significantly higher in the mortality group (all *P* < 0.05). Furthermore, the mortality group demonstrated significantly elevated levels of glucose, LDH, LAC, Scr, and UA (all *P* < 0.05). As shown in Supplementary Table S1, polytrauma patients (*n* = 160) had significantly lower PTS, lower albumin levels, and higher rates of transfusion and GCS score compared to isolated TBI patients (*n* = 129) (all *P* < 0.05).

### Feature predictor selection

**Figure 2:**
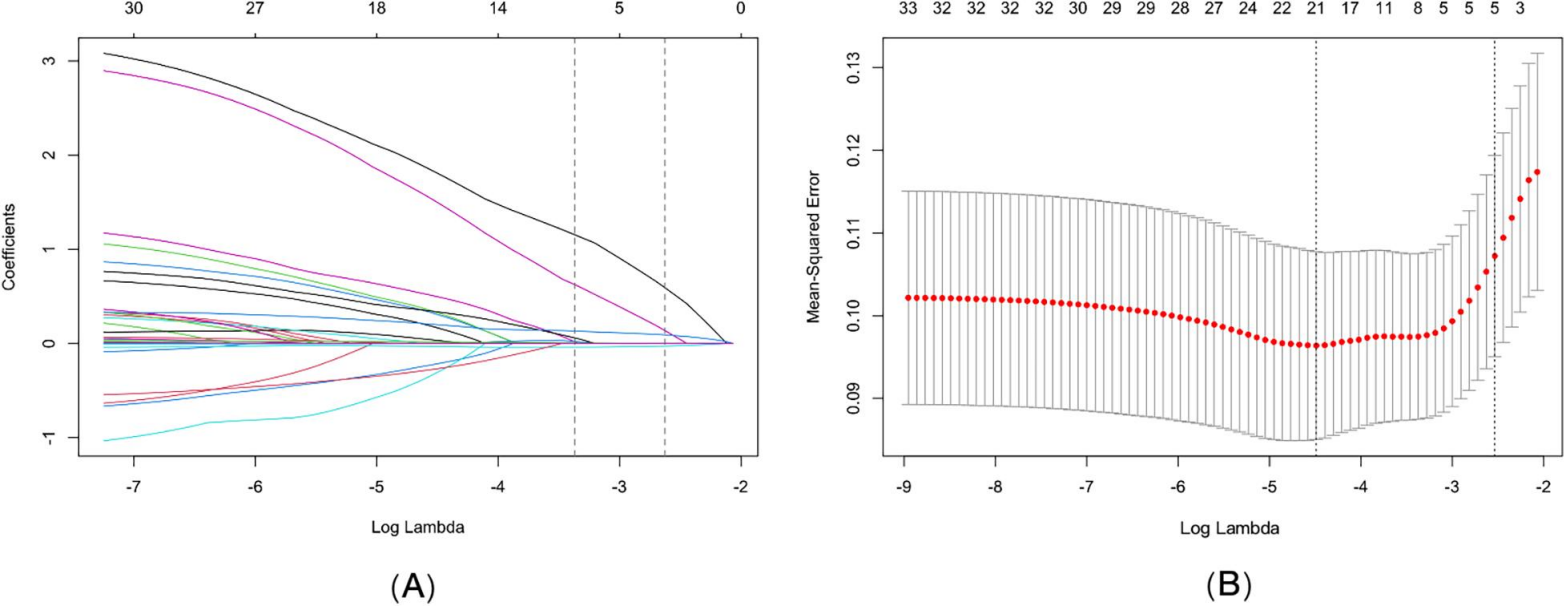
Selection of predictive features using LASSO regression. **(A)** LASSO coefficient profiles showing the relationship between log(lambda) and feature coefficients. As log(lambda) increases, coefficients shrink toward zero. **(B)** Tuning parameter (lambda) selection via 10-fold cross-validation. The left vertical dashed line indicates the lambda value with minimum mean-squared error, while the right dashed line represents the 1-SE criterion.

**Figure 3:**
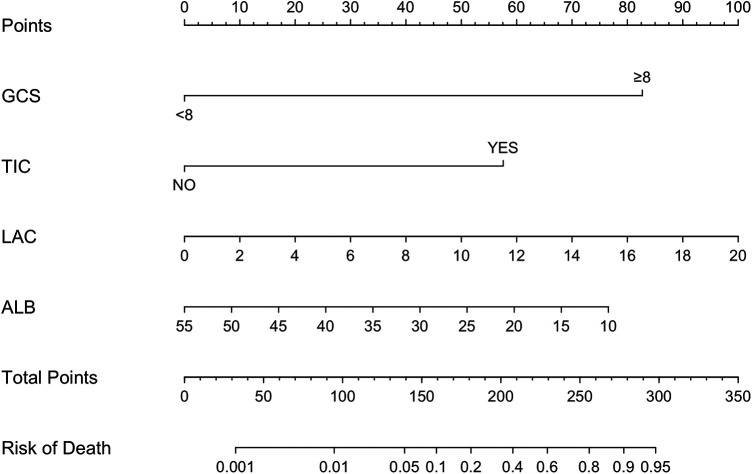
Clinical nomogram for predicting 30-day in-hospital mortality in children with msTBI.

The optimal combination of variables was identified through both LASSO regression and logistic regression analyses. Based on the results from the LASSO regression, five key variables with the highest relevance were selected and then further examined using a logistic regression model to explore their relationship with mortality outcomes and identify significant predictors (Figure 2 and Table 2). Among these, GCS score < 8 [OR = 18.49, 95% confidence interval (CI): 2.214, 154.438, *P* < 0.001], LAC (OR = 1.176, 95% CI: 1.017, 1.360, *P* < 0.05), albumin (OR = 0.942, 95% CI: 0.890, 0.998, *P* < 0.05), and TIC (OR = 8.076, 95% CI: 1.003, 65.043, *P* < 0.05) emerged as independent predictors of mortality. To visually represent the influence of each of these variables on the mortality outcome, a nomogram was developed, as shown in Figure 3. None of these variables violated the Variance Inflation Factor (VIF) criteria in the models. In the sensitivity analysis adjusting for polytrauma status shown in Supplementary Table S2, polytrauma was not independently associated with 30-day mortality (OR = 0.63, 95% CI 0.24–1.65, *P* = 0.352), while the other predictors remained significant.

**Table 2:** Multivariate logistic regression analysis after LASSO feature selection.

Variable	OR	95% CI	*P*
GCS score	18.49	(2.214,154.438)	0.007
LAC	1.176	(1.017,1.360)	0.028
Albumin	0.942	(0.890,0.998)	0.042
TIC	8.076	(1.003,65.043)	0.050
Scr	1.011	(0.990,1.032)	0.322

OR, odds ratios; CI, confidence intervals, GCS, Glasgow Coma Scale; LAC, lactic acid; TIC, trauma-induced coagulopathy; Scr, serum creatinine.

### Development and performance of the risk nomogram in the cohort

**Figure 4:**
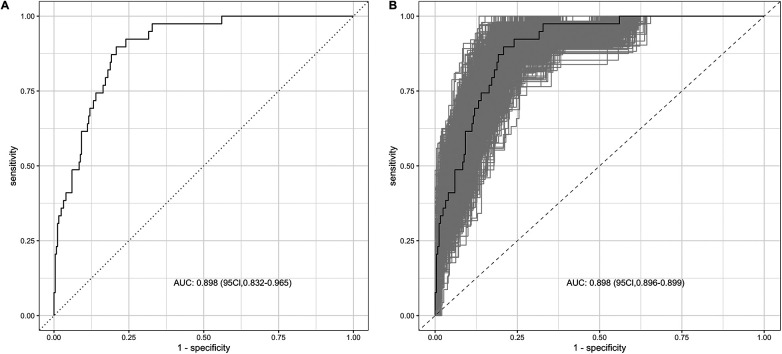
Performance of the clinical nomogram for predicting 30-day in-hospital mortality in children with msTBI. **(A)** Receiver Operating Characteristic (ROC) curve of the nomogram, demonstrating model discrimination with the calculated Area Under the Curve (AUC). **(B)** Summary of the ROC curve obtained from 1,000 bootstrap resampling iterations, illustrating internal validation stability and overfitting assessment.

As shown in Figure 4, the ROC curve for the logistic regression (LR) model exhibits an AUC of 0.898. Using the Youden index, the optimal cutoff probability for predicting 30-day mortality was 0.203, with a sensitivity of 92.3% and a specificity of 77.6%. The model underwent internal validation using the bootstrap method, with 1,000 resampling iterations. The average ROC curve obtained from these resampling iterations yielded an AUC of 0.898 (95% CI: 0.896, 0.899). This demonstrates the stability and robustness of the LR model across multiple resampling iterations.

**Figure 5:**
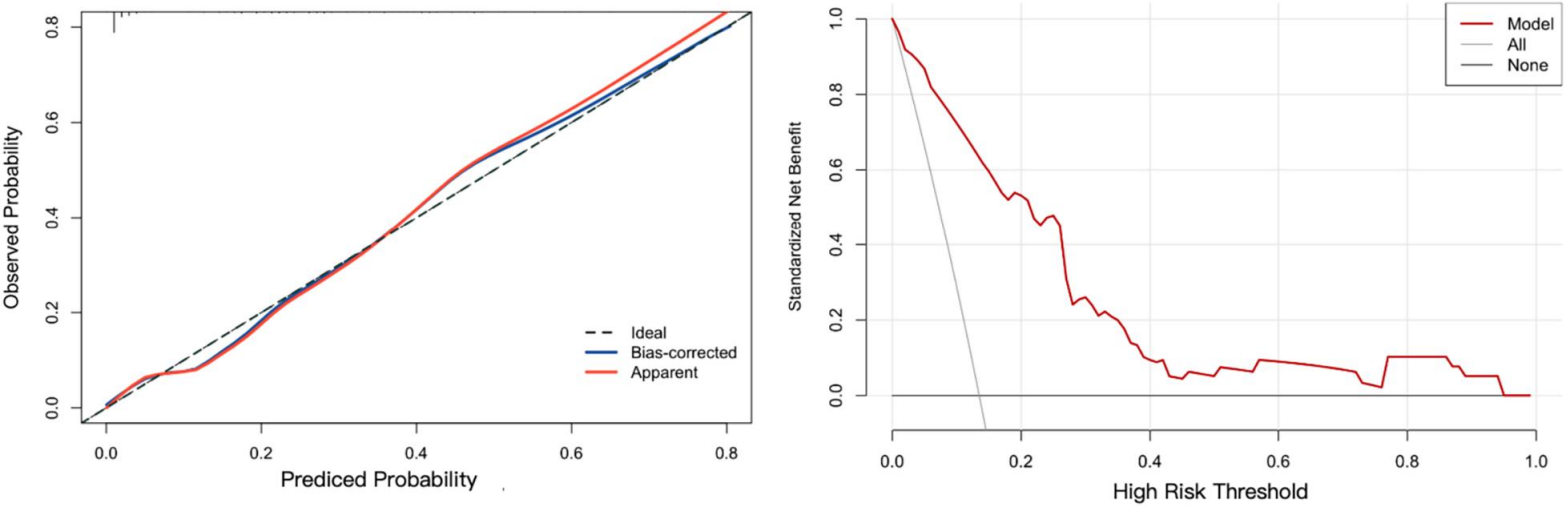
Calibration and Decision Curve Analysis (DCA) of the clinical nomogram for predicting 30-day in-hospital mortality in children with msTBI. Calibration plot comparing observed and predicted probabilities, showing the apparent, bias-corrected, and ideal lines. DCA of the model, demonstrating the standardized net benefit at different high-risk thresholds.

As shown in Figure 5, the calibration curve, generated using 1,000 bootstrap resampling with the apparent and bias-corrected lines, exhibited a slight deviation from the ideal line, indicating good consistency between predicted and observed outcomes. Furthermore, the *P*-value obtained from the Hosmer–Lemeshow test was 0.475, indicating no statistically significant difference, which further supported the model's calibration validity. Furthermore, based on the nomogram developed in this study, Decision Curve Analysis (DCA) demonstrated that the threshold probability ranged from 0 to 0.95.

## Discussion

In this study, we developed and validated a nomogram to predict 30-day in-hospital mortality in pediatric patients with msTBI. The logistic regression model demonstrated robust predictive performance, with an AUC of 0.898 (95% CI: 0.896, 0.899), both in the initial and bootstrap resampling validation. The key predictors of mortality identified were GCS score < 8, LAC, albumin, and TIC. Our findings suggest that these variables, particularly those associated with metabolic disturbances and neurological impairment, play a critical role in predicting the risk of mortality in TBI patients.

Previous studies have explored the prognostic factors for TBI and have developed nomograms with varying results. For example, Oearsakul et al. developed a nomogram for a 6-month follow-up outcome prediction in moderate or severe pediatric TBI with an AUC of 0.931 (11), while Zhu et al. proposed a model for predicting early mortality, achieving a concordance index of 0.918 (12). However, these studies were limited by small cohort sizes, potentially leading to overfitting and reduced generalizability. In contrast, our study utilized a larger cohort, improving the statistical power and reliability of the model. The predictive performance of our nomogram is comparable to those of previous models, yet our study addresses a critical gap by providing a prediction tool for 30-day in-hospital mortality, which has significant clinical implications for early intervention.

Currently, the GCS score is the most widely used tool for assessing the severity of TBI in both adult and pediatric populations (23). In a predictive model developed by Oearsakul et al., a GCS score of 3–8 was strongly associated with poor outcomes (OR: 16.07; 95% CI: 1.27, 202.42) (11). Similarly, Kan et al. reported that lower GCS scores were significantly correlated with adverse outcomes in children aged 2–16 years with TBI (5). However, as the standard GCS score relies on patient comprehension and response to verbal commands, its applicability in younger pediatric populations is limited. Therefore, in the present study, we employed the pGCS for children younger than 2 years, which is a validated and widely accepted modification of the standard GCS for preverbal children (15). Notably, GCS score <8 were significantly associated with 30-day in-hospital mortality in our cohort.

LAC is widely recognized as an indicator of metabolic stress, and its elevated levels have been consistently associated with poor outcomes in critically ill patients. In our cohort, LAC was significantly correlated with 30-day in-hospital mortality among children with msTBI. Consistent with our findings, Fu et al. demonstrated that higher LAC levels upon admission (OR: 1.189; 95% CI: 1.002–1.410) were independently predictive of mortality, suggesting that serum LAC can serve as a valuable early prognostic biomarker in pediatric TBI patients (24). This association may be explained by the disruption of cerebral energy metabolism following trauma, which contributes to secondary brain injury and worsens patient prognosis. Previous research has revealed that after TBI, lactate production in peripheral tissues rises, functioning as an alternative energy substrate for the injured brain. Moreover, surviving brain regions increase their uptake of lactate to compensate for impaired glucose metabolism. Elevated LAC may also reflect enhanced anaerobic glycolysis in response to cerebral hypoxia and reduced perfusion, both of which are key pathophysiological features of severe TBI (25, 26). In addition to increased production, elevated LAC levels can result from impaired clearance mechanisms, exceeding the body's metabolic capacity to eliminate it — a phenomenon often referred to as crossing the “anaerobic threshold” (27).

Hypoalbuminemia is frequently observed in critically ill neurosurgical patients, and emerging evidence suggests that lower serum albumin levels upon admission are significantly linked to increased mortality risk (28, 29). A retrospective study conducted in China further supported this association, indicating that initial serum albumin levels may serve as a useful prognostic marker for identifying pediatric patients with msTBI who are at higher risk of in-hospital death (30). The findings of our study align with these prior reports. One plausible explanation for this association is that reduced albumin levels may contribute to cerebral edema and elevated intracranial pressure due to compromised intravascular oncotic pressure (31, 32).

TIC is a distinct and early-onset coagulation disorder that occurs following traumatic injury, characterized by impaired hemostasis and an increased risk of bleeding (33). Our analysis identified TIC as a significant independent predictor of in-hospital mortality in children with msTBI. This is in agreement with the findings of Chong et al., who reported that early coagulopathy was independently associated with both increased mortality and unfavorable neurological outcomes in pediatric TBI patients (34). The potential mechanisms underlying this association include: (1) disruption of the blood–brain barrier, which allows brain-derived proinflammatory and coagulopathic mediators to enter the systemic circulation; and (2) a TBI-induced shift toward hypercoagulable and hyperfibrinolytic states, leading to ongoing intracranial and systemic bleeding (35). These alterations may aggravate secondary brain injury and contribute to poor outcomes.

As an intuitive clinical prediction tool, the nomogram translates complex regression equations into a visual scoring system, enabling bedside risk assessment by clinicians. Our nomogram incorporates readily available clinical variables including GCS, PTS, LAC, and ALB, which may help identify pediatric TBI patients at high risk of 30-day in-hospital mortality at an early stage. The nomogram could guide clinical decision-making such as intensified monitoring, early intervention, or optimized resource allocation. However, this study has several limitations that should be acknowledged. Firstly, it was conducted retrospectively at a single tertiary care center, which may introduce selection bias and limit the generalizability of the findings to broader pediatric populations. Although we employed rigorous statistical methods and internal validation through bootstrapping, external validation using multicenter or prospective cohorts is needed to confirm the robustness of the nomogram. Second, the lack of data on potentially important clinical variables, such as pharmacological interventions and intraoperative parameters, may have compromised the model's comprehensiveness. Additionally, the exclusion of patients with missing laboratory data may have introduced potential selection bias. Third, although the sensitivity analysis suggested that polytrauma status dose not materially affect the stability of our predictive model, the fundamental heterogeneity between isolated TBI and polytrauma patients remains uneliminated. Future studies should validate our nomogram separately in pure isolated TBI cohorts and in polytrauma cohorts to determine its generalizability across these distinct populations. Fourth, hemodilution from large-volume crystalloid resuscitation may have artifactually reduced admission albumin levels. Although sensitivity analyses (Supplementary Tables S3 and S4) suggested that albumin remained a robust predictor, we cannot completely exclude residual confounding. Future prospective studies are needed to validate our findings. Lastly, despite efforts to minimize missing data, laboratory and imaging variables collected at admission may vary in timing and accuracy, potentially introducing measurement bias.

## Conclusion

In this study, we developed and internally validated a nomogram for predicting 30-day in-hospital mortality in children with msTBI. The model, incorporating key clinical, radiological, and laboratory parameters, demonstrated strong discriminatory ability and good calibration. These findings suggest that the proposed nomogram may serve as a valuable tool for early risk stratification and clinical decision-making in pediatric TBI care. Future prospective and multicenter studies are warranted to externally validate its performance and assess its generalizability to other countries or ethnic groups.

## Data Availability

The raw data supporting the conclusions of this article will be made available by the authors, without undue reservation.

## References

[1] Ben Abdeljelil A Freire GC Yanchar N Turgeon AF Beno S Bérubé M. Pediatric moderate and severe traumatic brain injury: a systematic review of clinical practice guideline recommendations. J Neurotrauma. (2023) 40(21–22):2270–81. 10.1089/neu.2023.0149 37341019

[2] Dewan MC Mummareddy N Wellons JC 3rd Bonfield CM. Epidemiology of global pediatric traumatic brain injury: qualitative review. World Neurosurg. (2016) 91:497–509.e1. 10.1016/j.wneu.2016.03.045 27018009

[3] Rivara FP Koepsell TD Wang J Temkin N Dorsch A Vavilala MS. Incidence of disability among children 12 months after traumatic brain injury. Am J Public Health. (2012) 102(11):2074–9. 10.2105/ajph.2012.300696 22994196 PMC3477965

[4] Li Y Chen F Zhang J Li G Yang X Lu Q. Epidemiological characteristics of Chinese paediatric traumatic brain injury inpatients. Brain Inj. (2017) 31(8):1094–101. 10.1080/02699052.2017.1298004 28506081

[5] Kan CH Saffari M Khoo TH. Prognostic factors of severe traumatic brain injury outcome in children aged 2–16 years at a major neurosurgical referral centre. Malays J Med Sci. (2009) 16(4):25–33. 22135509 PMC3216137

[6] Cabrero Hernández M Iglesias Bouzas MI Martínez de Azagra Garde A Pérez Suárez E Serrano González A Jiménez García R. Early prognostic factors for morbidity and mortality in severe traumatic brain injury. Experience in a child polytrauma unit. Med Intensiva (Engl Ed). (2022) 46(6):297–304. 10.1016/j.medine.2022.04.013 35562275

[7] Rosario BL Horvat CM Wisniewski SR Bell MJ Panigrahy A Zuccoli G. Presenting characteristics associated with outcome in children with severe traumatic brain injury: a secondary analysis from a randomized, controlled trial of therapeutic hypothermia. Pediatr Crit Care Med. (2018) 19(10):957–64. 10.1097/pcc.0000000000001676 30067578 PMC6170689

[8] Alharfi IM Stewart TC Kelly SH Morrison GC Fraser DD. Hypernatremia is associated with increased risk of mortality in pediatric severe traumatic brain injury. J Neurotrauma. (2013) 30(5):361–6. 10.1089/neu.2012.2410 23057958

[9] Hochstadter E Stewart TC Alharfi IM Ranger A Fraser DD. Subarachnoid hemorrhage prevalence and its association with short-term outcome in pediatric severe traumatic brain injury. Neurocrit Care. (2014) 21(3):505–13. 10.1007/s12028-014-9986-7 24798696

[10] Cerasa A Tartarisco G Bruschetta R Ciancarelli I Morone G Calabrò RS. Predicting outcome in patients with brain injury: differences between machine learning versus conventional statistics. Biomedicines. (2022) 10(9):2267. 10.3390/biomedicines10092267 PMC9496389 36140369

[11] Oearsakul T Tunthanathip T. Development of a nomogram to predict the outcome of moderate or severe pediatric traumatic brain injury. Turk J Emerg Med. (2022) 22(1):15–22. 10.4103/2452-2473.336107 35284689 PMC8862794

[12] Zhu P Hussein NM Tang J Lin L Wang Y Li L. Prediction of early mortality among children with moderate or severe traumatic brain injury based on a nomogram integrating radiological and inflammation-based biomarkers. Front Neurol. (2022) 13:865084. 10.3389/fneur.2022.865084 35669876 PMC9163313

[13] Steyerberg EW Uno H Ioannidis JPA Van Calster B Ukaegbu C Dhingra T. Poor performance of clinical prediction models: the harm of commonly applied methods. J Clin Epidemiol. (2017) 98:S0895435617307886. 10.1016/j.jclinepi.2017.11.013 29174118

[14] Tepas JJ III Mollitt DL Talbert JL Bryant M. The pediatric trauma score as a predictor of injury severity in the injured child. J Pediatr Surg. (1987) 22(1):14–8. 10.1016/S0022-3468(87)80006-4 3102714

[15] Marcin JP Pollack MM. Triage scoring systems, severity of illness measures, and mortality prediction models in pediatric trauma. Crit Care Med. (2002) 30(11):S457–67. 10.1097/00003246-200211001-00011 12528788

[16] Maas AI Hukkelhoven CW Marshall LF Steyerberg EW. Prediction of outcome in traumatic brain injury with computed tomographic characteristics: a comparison between the computed tomographic classification and combinations of computed tomographic predictors. Neurosurgery. (2005) 57(6):1173–82. discussion -82. 10.1227/01.neu.0000186013.63046.6b 16331165

[17] Liesemer K Riva-Cambrin J Bennett KS Bratton SL Tran H Metzger RR. Use of rotterdam CT scores for mortality risk stratification in children with traumatic brain injury. Pediatr Crit Care Med. (2014) 15(6):554–62. 10.1097/pcc.0000000000000150 24751786 PMC4087067

[18] Talari HR Hamidian Y Moussavi N Fakharian E Abedzadeh-Kalahroudi M Akbari H. The prognostic value of rotterdam computed tomography score in predicting early outcomes among children with traumatic brain injury. World Neurosurg. (2019) 125:e139–e45. 10.1016/j.wneu.2018.12.221 30677579

[19] Pasricha SR Rogers L Branca F Garcia-Casal MN. Measuring haemoglobin concentration to define anaemia: wHO guidelines. Lancet. (2024) 403(10440):1963–6. 10.1016/s0140-6736(24)00502-6 38493792

[20] Deshpande SJ Tsang HC Phuongs J Hasan R Liu Z Stansbury LG. Trauma-induced coagulopathy across age pediatric groups: a retrospective cohort study evaluating testing and frequency. Paediatr Anaesth. (2025) 35(1):57–65. 10.1111/pan.15024 39435581

[21] de Simone G Mancusi C Hanssen H Genovesi S Lurbe E Parati G. Hypertension in children and adolescents. Eur Heart J. (2022) 43(35):3290–301. 10.1093/eurheartj/ehac328 35896123

[22] Peduzzi P Concato J Kemper E Holford TR Feinstein AR. A simulation study of the number of events per variable in logistic regression analysis. J Clin Epidemiol. (1996) 49(12):1373–9. 10.1016/s0895-4356(96)00236-3 8970487

[23] Marshall LF Becker DP Bowers SA Cayard C Eisenberg H Gross CR. The national traumatic coma data bank. Part 1: design, purpose, goals, and results. J Neurosurg. (1983) 59(2):276–84. 10.3171/jns.1983.59.2.0276 6345728

[24] Fu YQ Bai K Liu CJ. The impact of admission serum lactate on children with moderate to severe traumatic brain injury. PLoS One. (2019) 14(9):e0222591. 10.1371/journal.pone.0222591 31536567 PMC6752785

[25] Özel A İlbeği EN Yüce S. Predictive value of initial lactate levels for mortality and morbidity in critically ill pediatric trauma patients: a retrospective study from a turkish pediatric intensive care unit. Acute Crit Care. (2025) 40(1):87–94. 10.4266/acc.003528 39978951 PMC11924349

[26] Glenn TC Martin NA Horning MA McArthur DL Hovda DA Vespa P. Lactate: brain fuel in human traumatic brain injury: a comparison with normal healthy control subjects. J Neurotrauma. (2015) 32(11):820–32. 10.1089/neu.2014.3483 25594628 PMC4530406

[27] Myers J Ashley E. Dangerous curves. A perspective on exercise, lactate, and the anaerobic threshold. Chest. (1997) 111(3):787–95. 10.1378/chest.111.3.787 9118720

[28] Morotti A Marini S Lena UK Crawford K Schwab K Kourkoulis C. Significance of admission hypoalbuminemia in acute intracerebral hemorrhage. J Neurol. (2017) 264(5):905–11. 10.1007/s00415-017-8451-x 28283821 PMC7436338

[29] Sung J Bochicchio GV Joshi M Bochicchio K Scalea TM. Admission serum albumin is predicitve of outcome in critically ill trauma patients. Am Surg. (2004) 70(12):1099–102. 10.1177/000313480407001214 15663053

[30] Luo H, Fu Y, You C. Comparison of admission serum albumin and hemoglobin as predictors of outcome in children with moderate to severe traumatic brain injury: a retrospective study. *Medicine*. (2019) 98(51):e18562. 10.1097/MD.0000000000017806 PMC6946495 31689863

[31] Baltazar GA Pate AJ Panigrahi B LaBoy S Prosniak R Mody A. Malnutrition as measured by albumin and prealbumin on admission is associated with poor outcomes after severe traumatic brain injury. Am Surg. (2015) 81(2):E61–3. 10.1177/000313481508100208 25642858

[32] Wang R He M Qu F Zhang J Xu J. Lactate albumin ratio is associated with mortality in patients with moderate to severe traumatic brain injury. Front Neurol. (2022) 13:662385. 10.3389/fneur.2022.662385 35432157 PMC9011050

[33] Kornblith LZ Moore HB Cohen MJ. Trauma-induced coagulopathy: the past, present, and future. J Thromb Haemost. (2019) 17(6):852–62. 10.1111/jth.14450 30985957 PMC6545123

[34] Chong SL Ong GY Zheng CQ Dang H Ming M Mahmood M. Early coagulopathy in pediatric traumatic brain injury: a pediatric acute and critical care medicine Asian network (PACCMAN) retrospective study. Neurosurgery. (2021) 89(2):283–90. 10.1093/neuros/nyab157 33913493

[35] Zhang J Zhang F Dong JF. Coagulopathy induced by traumatic brain injury: systemic manifestation of a localized injury. Blood. (2018) 131(18):2001–6. 10.1182/blood-2017-11-784108 29507078 PMC5934798

