# Supplementary material for: Development and validation of a clinical nomogram for predicting 30-day in-hospital mortality in children with moderate-to-severe traumatic brain injury
Source: Front Pediatr. 2026 Jun 8;14:1754122. doi: 10.3389/fped.2026.1754122 (PMC13283873; doi:10.3389/fped.2026.1754122)
Supplement: Supplementary file 1 [file Table1.docx]

**Table S1.** Demographic and clinical characteristics of children with msTBI.

| Character | Total (n = 289) | **Isolated TBI group** | **Polytrauma group** | *P* |
| --- | --- | --- | --- | --- |
|  |  | (n = 129) | (n = 160) |  |
| Gender, n (%) |  |  |  | **0.694** |
| Female | 101 (35) | 43 (33) | 58 (36) |  |
| Male | 188 (65) | 86 (67) | 102 (64) |  |
| Age (years) | 5.17 (2.75, 9.33) | 4.83 (1.83, 8.42) | 5.33 (3.33, 9.86) | **0.013** |
| Weight (kg) | 19 (14, 30) | 17.75 (12, 29) | 20 (15, 33.88) | **0.018** |
| Time from injury to hospital (hours) | 3 (2, 4) | 3 (2, 4) | 3 (2, 4) | 0.357 |
| Mechanism of injury, n (%) |  |  |  | **< 0.001** |
| Traffic accident | 138 (48) | 47 (36) | 91 (57) |  |
| Fall off | 132 (46) | 66 (51) | 66 (41) |  |
| Other | 19 (7) | 16 (12) | 3 (2) |  |
| PTS, n (%) |  |  |  | **< 0.001** |
| ≤ 5 | 109 (38) | 24 (19) | 85 (53) |  |
| ＞5 | 180 (62) | 105 (81) | 75 (47) |  |
| GCS score, n (%) |  |  |  | **< 0.001** |
| < 8 | 138 (48) | 77 (60) | 61 (38) |  |
| ≥ 8 | 151 (52) | 52 (40) | 99 (62) |  |
| Blood transfusion, n (%) | 148 (51) | 53 (41) | 95 (59) | **0.003** |
| Neurosurgical operation, n (%) | 155 (54) | 52 (40) | 103 (64) | **< 0.001** |
| Skull fractures, n (%) | 212 (73) | 92 (71) | 120 (75) | 0.569 |
| Epidural hematoma, n (%) | 110 (38) | 56 (43) | 54 (34) | 0.119 |
| Subdural hematoma, n (%) | 92 (32) | 44 (34) | 48 (30) | 0.536 |
| Subarachnoid hemorrhage, n (%) | 164 (57) | 60 (47) | 104 (65) | **0.002** |
| Intraventricular hemorrhage, n (%) | 30 (10) | 9 (7) | 21 (13) | 0.131 |
| Diffuse axonal injury, n (%) | 35 (12) | 6 (5) | 29 (18) | **< 0.001** |
| Brain contusion, n (%) | 118 (41) | 49 (38) | 69 (43) | 0.445 |
| Long bone fractures, n (%) | 57 (20) | 0 (0) | 57 (36) | **< 0.001** |
| Pelvic fractures, n (%) | 39 (13) | 0 (0) | 39 (24) | **< 0.001** |
| Chest trauma, n (%) | 135 (47) | 0 (0) | 135 (84) | **< 0.001** |
| Abdominal organ injury, n (%) |  |  |  | **< 0.001** |
| NO | 224 (78) | 118 (100) | 106 (66) |  |
| Contusion | 51 (18) | 0 (0) | 51 (32) |  |
| Injury by rupture | 13 (4) | 0 (0) | 13 (8) |  |
| TIC, n (%) | 163 (56) | 56 (43) | 107 (67) | **< 0.001** |
| Anemia, n (%) | 212 (73) | 88 (68) | 124 (78) | 0.101 |
| Hypotension, n (%) | 122 (48) | 43 (42) | 79 (52) | 0.14 |
| Glucose (mmol/L ) | 7.5 (6.1, 10.4) | 7.35 (6.03, 9.43) | 7.95 (6.2, 11.17) | 0.132 |
| WBC (×10⁹/L) | 17.72 (12.9, 22.77) | 16.51 (12.7, 21.58) | 18.2 (13.22, 23.69) | 0.068 |
| LDH (U/L) | 573.2 (375.5, 998.2) | 400.5 (346.08, 605.32) | 773.5 (513.6, 1285.53) | **< 0.001** |
| LAC (mmol/L) | 2.7 (1.7, 4) | 2.7 (1.9, 3.65) | 2.65 (1.52, 4.27) | 0.895 |
| Albumin (g/L) | 38.4 (33.55, 42.2) | 39 (34.92, 42.7) | 29.55 (24.38, 38.25) | **< 0.001** |
| BUN (mg/dL ) | 4.63 (3.77, 5.77) | 4.43 (3.62, 5.61) | 4.7 (4.05, 5.98) | **< 0.001** |
| Scr (μmol/L ) | 31 (23, 41.68) | 27 (21, 35) | 35 (26, 45.5) | **< 0.001** |
| UA (μmol/L ) | 284.55 (238.4, 357.65) | 258.4 (217.35, 307.2) | 325.8 (261.6, 393.75) | **< 0.001** |

**Abbreviation:** **PTS, Pediatric Trauma Score; GCS, Glasgow Coma Scale; TIC, trauma-induced coagulopathy; WBC, white blood cells; LDH, lactate dehydrogenase; LAC, lactic acid; BUN, blood urea nitrogen; Scr, serum creatinine; UA, uric acid。**

**Table S2**Multivariable logistic regression analysis adjusting for polytrauma status

| Variable | OR | 95% CI | P |
| --- | --- | --- | --- |
| GCS | 27.19 | (2.68, 275.6) | 0.005 |
| TIC | 9.28 | (1.15, 74.7) | 0.036 |
| LAC | 1.24 | (1.07, 1.43) | 0.003 |
| Albumin | 0.94 | (0.88, 0.99) | 0.030 |
| Polytrauma | 0.69 | (0.27, 1.76) | 0.433 |

**Abbreviation:** **OR, odds ratios; CI, confidence intervals, GCS, Glasgow Coma Scale; LAC, lactic acid; TIC, trauma-induced coagulopathy;**

**Table S3.** Albumin levels stratified by blood transfusion status

| **Blood Transfusion status** | **Group** | **n** | **Albumin (g/L)** | ***P*** |
| --- | --- | --- | --- | --- |
| No transfusion | Survival group | 132 | 41.15 (37.80, 43.90) | 0.003 |
|  | Mortality group | 9 | 27.50 (21.30, 39.68) |  |
| Transfusion | Survival group | 118 | 35.88 ± 6.09 | 0.015 |
|  | Mortality group | 30 | 31.25 ± 9.47 |  |

Data are presented as median (IQR) for the no transfusion group and mean ± SD for the transfusion group. P values were calculated using the Wilcoxon rank-sum test (no transfusion group) or Student's t-test (transfusion group).

### **Table S4.** Multivariable logistic regression model adjusting for blood transfusion and hypotension

| Variable | OR | 95% CI | *P* |
| --- | --- | --- | --- |
| GCS | 30.22 | (2.96, 308.5) | 0.004 |
| TIC | 11.38 | (1.36, 95.0) | 0.025 |
| LAC | 1.29 | (1.10, 1.51) | 0.002 |
| Albumin | 0.94 | (0.89, 1.00) | 0.043 |
| Hypotension | 0.47 | (0.18, 1.21) | 0.117 |
| Blood transfusion | 0.79 | (0.28, 2.22) | 0.652 |

### **Abbreviation: OR, odds ratios; CI, confidence intervals, GCS, Glasgow Coma Scale; LAC, lactic acid; TIC, trauma-induced coagulopathy;**
